# Effect of *Phlebotomus papatasi* on the fitness,infectivity and antimony-resistance phenotype of antimony-resistant *Leishmania major* Mon-25

**DOI:** 10.1016/j.ijpddr.2024.100554

**Published:** 2024-06-24

**Authors:** Nalia Mekarnia, Benallal Kamal-Eddine, Jovana Sádlová, Barbora Vojtková, Aurélie Mauras, Nicolas Imbert, Maryline Longhitano, Zoubir Harrat, Petr Volf, Philippe M. Loiseau, Sandrine Cojean

**Affiliations:** a UR 7510 ESCAPE - USC Anses, School of Pharmacy, Université de Reims Champagne-Ardenne, Pôle Santé, 51100 Reims, France; b UMR 8076 BioCIS, CNRS, Université Paris-Saclay, 91400, Orsay, France; c Department of Parasitology, Faculty of Sciences, Charles University, Vinicna 7, Prague, Czech Republic; d Laboratory of Arboviruses and Emergent Viruses, Institut Pasteur d’Algérie, 16047, Algiers, Algeria; e UMR BIPAR, Laboratory of Animal Health, Anses, INRAe, EnvA, 94700, Maisons-Alfort, France; f National Malaria Reference Center, AP-HP, Hôpital Bichat Claude Bernard, 75018, Paris, France; g Algerian Academy for Science and Technology, Villa Rais Hamidou, Chemin Omar Kachkar, El Madania, Algiers, Algeria

**Keywords:** *Leishmania major*, *Phlebotomus papatasi,* experimental infection, Fitness, Virulence, Antimony resistance

## Abstract

*Leishmania major* is responsible for zoonotic cutaneous leishmaniasis. Therapy is mainly based on the use of antimony-based drugs; however, treatment failures and illness relapses were reported. Although studies were developed to understand mechanisms of drug resistance, the interactions of resistant parasites with their reservoir hosts and vectors remain poorly understood. Here we compared the development of two *L. major* MON-25 trivalent antimony-resistant lines, selected by a stepwise *in vitro* Sb(III)-drug pressure, to their wild-type parent line in the natural vector *Phlebotomus papatasi.* The intensity of infection, parasite location and morphological forms were compared by microscopy. Parasite growth curves and IC_50_ values have been determined before and after the passage in *Ph. papatasi*. qPCR was used to assess the amplification rates of some antimony-resistance gene markers. In the digestive tract of sand flies, Sb(III)-resistant lines developed similar infection rates as the wild-type lines during the early-stage infections, but significant differences were observed during the late-stage of the infections. Thus, on day 7 p. i., resistant lines showed lower representation of heavy infections with colonization of the stomodeal valve and lower percentage of metacyclic promastigote forms in comparison to wild-type strains. Observed differences between both resistant lines suggest that the level of Sb(III)-resistance negatively correlates with the quality of the development in the vector. Nevertheless, both resistant lines developed mature infections with the presence of infective metacyclic forms in almost half of infected sandflies. The passage of parasites through the sand fly guts does not significantly influence their capacity to multiply *in vitro*. The IC_50_ values and molecular analysis of antimony-resistance genes showed that the resistant phenotype of Sb(III)-resistant parasites is maintained after passage through the sand fly. Sb(III)-resistant lines of *L. major* MON-25 were able to produce mature infections in *Ph. papatasi* suggesting a possible circulation in the field using this vector.

## Highlights

• Antimony-resistant *Leishmania major* developed infection in *Phlebotomus papatasi.*
• Resistant lines showed differences with wild-types along the infection kinetics.
• The resistant phenotype of *L. major* is maintained after passage through a sand fly.
• Resistant-*Leishmania* passage in the vector maintained their *vitro* multiplication.
• The vector could promote circulation of antimony-resistant *L. major* in the field.

## Graphical abstract

**Figure:**
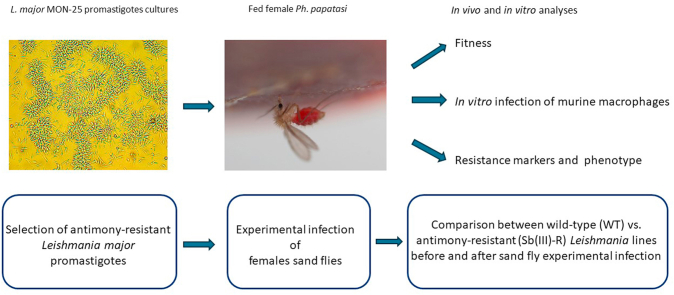

## 1 Introduction

Leishmaniases are regarded as neglected vector-borne infectious diseases caused by flagellated protozoan belonging to the *Trypanosomatidae* family and *Leishmania* genus. According to the WHO, 200 countries and territories have reported cases of leishmaniasis (World Health Organization, 2020). Nowadays, more than 1 billion people live in areas considered endemic for leishmaniasis and are at risk of infection. Annually, 30,000 estimated new cases of visceral leishmaniasis (VL) are reported and more than 1 million new cases of cutaneous leishmaniasis (CL) occur (World Health Organization, 2023).

Algeria is ranked second endemic country to CL worldwide after Afghanistan (World Health Organization, 2022). The disease is caused by three *Leishmania* species with different vectors and reservoir hosts *i) L. major* MON-25 transmitted by *Ph. papatasi* (Izri et al., 1992) and wild rodents *Meriones shawi* and *Psammomys obesus* as the main and proven reservoirs (Belazzoug, 1983, 1986), *ii) L. tropica* (reported as *L. killicki* MON-301) transmitted by *Ph. sergenti* with the natural reservoir *Ctenodactylus gundi* (Boubidi et al., 2011; Jaouadi et al., 2011) and *iii) L. infantum* MON-24 transmitted by *Ph. (Laroussius) perfiliewi* and the dog as the main reservoir (Izri and Belazzoug, 1993; Benikhlef et al., 2004).

During the past 20 years, the emergence of clinical resistant *Leishmania* parasites against the available antileishmanial drugs such as pentavalent (Sb(V)) antimonials, miltefosine (MIL) and paromomycin has dangerously increased worldwide whereas clinical resistant cases to amphotericin B (AmB) remain rarely reported (Ponte-Sucre et al., 2017). In Algeria, meglumine antimoniate (Glucantime®) remains the first-line drug used for both VL and CL (Sereno et al., 2012). Unfortunately, treatment failures have been reported in several regions of Algeria, and drug resistance observed in patients treated with Glucantime® remains a major issue in antileishmanial chemotherapy (Ponte-Sucre et al., 2017; Eddaikra et al., 2018). Because antimonials are the primary antileishmanial drugs used in most developing countries, a comprehension of the fitness cost associated with the acquisition of drug resistance phenomenon is important to design efficient control tools and management of leishmaniasis (Ait-Oudhia et al., 2011). Moreover, various factors may play a role in the selection of Sb-resistant lines such as host genetic background and immunological responses to treatment, drug quality, the non-respect of therapy and recommended doses, and the intrinsic drug-susceptibility of *Leishmania* species (Ponte-Sucre et al., 2013; Eddaikra et al., 2018). In addition, *Leishmania* parasites are known to be essentially clonal with genetic exchanges taking place in invertebrate hosts (Akopyants et al., 2009) which may result in increasing the fitness of the offspring (Volf et al., 2007) and selection/elimination of some clones under the bottleneck phenomena (Bussotti et al., 2023). Thus, the spread of a drug-resistant phenotype in the field depends on the capacity of resistant parasites to survive inside the different hosts (reservoir and vector) and to be successfully transmitted between hosts during the parasite life-cycle. So far, the mechanisms by which resistant lines are maintained and transmitted between mammalian reservoirs and vectors remain poorly understood and relatively few studies focused on this important subject (Seblova et al., 2014; Van Bockstal et al., 2019, 2020; Hendrickx et al., 2020a, Hendrickx et al., 2020b).

In this context with the objective to better understand the spreading of antimony-resistant *Leishmania* parasites in the field, this study aimed to assess the impact of Sb(III)-resistance on the development of *L. major* in the competent sand fly vector. For this purpose, females *Ph. papatasi* were experimentally infected with two susceptible wild-type strains (WT) and two Sb(III)-resistant lines differing in resistance levels. Parasite loads, morphological forms and location of parasites in various parts of the sand fly digestive tracts were compared *in vivo* and parasites isolated from infected guts of female sand flies were studied *in vitro* to evaluate several biological parameters: growth capacity, infection rate and parasite load in macrophages, parasites drug susceptibility and the expression rate of targeted genes as resistance markers.

## 2 Materials and methods

### 2.1 Parasite strains and cultures

Two virulent wild-type strains were used to run this study. The WT *Leishmania major* MON-25 strain MHOM/DZ/09/LIPA100/09/MON-25 (LIPA-WT) was isolated from a patient living in M'Sila province in Algeria and maintained in culture at Institut Pasteur d’Algérie MHOM/LY/87/CS3 (Liby) was isolated in Prague from a patient coming back from Libya. These two lines were cultured in complete M199 medium (Sigma) containing 10% heat-inactivated FCS (Gibco) supplemented with 1% BME vitamins (Basal Medium Eagle, Sigma), 2% sterile human urine and 250 μg/mL amikacin (Amikin, Bristol-Myers Squibb) for sand fly experiments in Prague.

### 2.2 Selection of Sb(III)-resistant lines

*Leishmania*-resistant lines were selected *in vitro* at PARACHEM laboratory (UMR CNRS 8076 BioCIS at Université Paris-Saclay) by discontinuous stepwise drug pressure on the LIPA-WT strain using antimony potassium tartrate (Sb(III)) (Sigma-Aldrich, Saint Quentin Fallavier, France) at different concentrations (0.1, 0.5, 1, 2, 5, 10 mM) according to Hendrickx et al. (2012) with slight modifications. Parasites (10^6^ promastigotes/mL) were cultured in duplicate in M199 medium (ThermoFisher, Villebon sur Yvette, France) supplemented with 40 mM HEPES, 100 μM adenosine, 0.5 mg/mL haemin, and 10% heat-inactivated fetal calf serum (FCS) (Life Technologies-Gibco, Mexico origin, France). Drug-pressure selection was assessed in TPP® 24-wells flat-bottom plates at 26 °C in a dark environment. The assays were carried out in duplicate for each drug concentration and the parasites under drug pressure were incubated at 26 °C overnight until the appearance of clusters. The promastigotes which had survived the action of the first concentration (0.1 mM) of Sb(III) were transferred into 5 mL of free-drug complete M199 medium and then incubated at 26 °C. Once the promastigotes have reached their logarithmic phase of growth, the drug pressure resumed starting from the last value of the maximum concentration plateau obtained previously (0.1 mM), gradually increasing the concentrations (0.2, 0.25, 0.35, 0.4, 0.45 mM) until reaching a maximum concentration value at 0.5 mM, to get the intermediate resistant line (SbIIIR-0.5 mM). A second level of resistance was obtained by increasing the doses of Sb(III) starting from the last maximum dose of 0.5 mM until reaching a maximum dose of 5.5 mM (SbIIIR-5.5 mM). Parasite growth was determined using SYBR® Green I asymmetrical cyanine dye (Invitrogen, Villebon sur Yvette, France), the incorporation of SYBR® Green I in parasite DNA was measured using the Master epRealplex cycler® (Eppendorf, Montesson, France). The results were expressed as IC_50_ values that were determined by a nonlinear regression using ICEstimator website 1.2 version: ICE. Method Introduction (antimalarial-icestimator.net). Fluorescence data were compared to those acquired from the range with different parasite densities. Miltefosine (HePC: hexadecyl-phosphorylcholine) was used as a reference compound. The antileishmanial activity was expressed as IC_50_ in μM (concentration of drug inhibiting the parasite growth by 50%, in comparison to the controls treated with the excipient only).

### 2.3 Sand flies and sand fly infections

*Phlebotomus papatasi* colony was maintained in the Department of Parasitology at Charles University of Prague, under standard conditions at 26 °C, 50% sucrose and 14 h light/10 h dark photoperiod as previously described by Volf and Volfova (2011). Parasites (two WT strains (LIPA-WT and Liby) and two resistant lines derived from the LIPA-WT strain (SbIIIR-0.5 mM and SbIIIR-5.5 mM) at the logarithmic phase of culture were washed twice in sterile saline (NaCl) and re-suspended in heat-inactivated rabbit blood at a final concentration of 10^6^ promastigotes/mL. 5–9 days old *Ph. papatasi* females were fed through a chicken-skin membrane containing a suspension of promastigote parasites in a dark room for 1 h at 26 °C according to Sádlová et al. (2017)*.* Unfed females were removed, and the engorged ones were maintained under the same conditions as the colony. The experiments were performed in triplicate. On day 2 and 7 post-infection (p.i.), fifteen females from each cage were dissected in a drop of saline under stereomicroscope. The intensity and location of infection in the digestive tracts of the sand flies were examined under the light microscope (Olympus DP70). Parasite load was considered light for <100 parasites per gut, moderate for 100–1000 parasites per gut, and heavy for more than 1000 parasites per gut, according to Myskova et al. (2008). The average number of parasites present in sand fly digestive tracts were estimated and represented graphically with Microsoft® Excel 2016 software. Differences in infection intensities, the percentage of morphological forms found in infected sand flies and parasite localization in digestive tracts were applied to Chi-square (χ2) tests. The statistical differences are considered significant if *P_*value*_<0.05 and highly significant if **P_*value*_*<*0.01.

### 2.4 Morphology of parasites in sand fly

Morphology of L. *major* parasites was evaluated from gut-smears of infected female sand flies dissected on day 7 p. i. Five gut-smears were used per each line and day of dissection. Ten random observation fields containing parasites were observed and photographed in each smear under the light microscope (Olympus DP70). The body length, body width and flagellum length were measured using the ImageJ® software (NIH, Bethesda, Maryland, USA). The morphological stages of the parasites were determined according to the established criteria of Sádlová et al*.* 2010; (2017) and Rogers et al*.* (2002): short promastigotes (SP) body length <14 μm; elongated nectomonads (EN) body length ⩾14 μm; metacyclic promastigotes (MC) flagellum two times > body length and a body length <14 μm.

### 2.5 Parasite recovery from sand flies

*L. major* LIPA-WT and Sb(III)-resistant lines were isolated from infected *Ph. papatasi* females dissected individually at day 7 p. i in sterile saline. The guts containing parasites were inoculated individually into SNB-9 blood agar slopes (Diamond and Herman, 1954) overlaid with M199 medium (the same used for cultivation of promastigotes, supplemented additionally with Fluorocytosine 100 μg/ml). After three days, if there was no contamination, the cultures were frozen.

### *2.6* L. major *Mon-25 in vitro fitness*

The fitness of the three *L. major* lines (LIPA-WT and both Sb(III)-resistant lines) was estimated by following up the growth curves of 2 × 10^6^ parasites/mL promastigotes from the stationary phase of development inoculated in a suspension 10 mL of M199 complete medium and incubated at 26 °C. To avoid any bias, parasites were counted in duplicate (2 flasks of sub-cultures/parasites line) by two independent manipulators than the data were pooled. The parasite density was assessed every 24 h (24–120 h). Parasites growth curves were represented using GraphPad Prism software version 7.05 (GraphPad Software Inc.). Two-way ANOVA followed by *Dunnett's* multiple comparisons test was used for multigroups comparison. Results were expressed as mean ± SD and considered significant if *P*_*value*_< 0.05.

### 2.7 Evaluation of Sb(III)-susceptibility

Sb(III)-susceptibility assays were performed on *L. major* MON-25 (LIPA-WT) and both antimony-resistant lines. Promastigotes (10^6^ parasites/mL) isolated from the infected sand flies were cultured in sterile 96-Well flat-bottomed microplates (Thermo Scientific™) at 26 °C in a dark environment. The assays were carried out in duplicate for each *Leishmania* line. After 72 h of incubation, parasite growth was determined using SYBR® Green I asymmetrical cyanine dye (Invitrogen, Villebon sur Yvette, France). The incorporation of SYBR® Green I in the DNA of parasites was measured using the Master epRealplex cycler® (Eppendorf, Montesson, France). The results were expressed as IC_50_ values that were determined by a nonlinear regression using IC Estimator website 1.2 version as previously described (section *2.2. Selection of Sb(III)-resistant lines*).

### 2.8 Infection of macrophages by *L. major* LIPA-WT and Sb(III)-resistant lines

The murine monocytes/macrophages cell line RAW 264.7 was maintained on T75 sterile culture flasks with filter, in Dulbecco's Modified Eagle's liquid Medium (DMEM) (ThermoFisher, Villebon sur Yvette, France) supplemented with 10% heat-inactivated FCS and 1% Penicillin/Streptomycin antibiotics. The cultures were incubated in the dark at 37 °C under 5% CO2 and used for the experiments when cell's confluence reached 80%. To run the assay, sterile glass cover slips were placed at the bottom of 12-wells flat bottom plate (Thermo Scientific™). RAW 264.7 cell lines were seeded into the 12-wells plate at a density of 500,000 cells/mL in 100 μL of complete DMEM in each well and then incubated in a dark environment for 24 h at 37 °C under 5% CO2 to plate the cells onto the cover slips. In parallel, parasite suspensions of LIPA-WT, SbIIIR-0.5 mM and SbIIIR-5.5 mM were cultivated to yield a rate of 5x106 parasites/mL in complete DMEM liquid medium at 37 °C. After incubation of RAW 264.7 cells overnight, the DMEM was replaced with 100 μL of fresh DMEM containing a suspension of *L. major* parasites prepared at a concentration of 10^6^ parasites/mL. The experiment was performed in triplicate for each parasite line. Infection kinetics was carried out at 6 h, 9 h, 24 h, 48 h and 72 h after infection; the infection rate (percentage of macrophage infected) as well as the parasite load per macrophage were assessed. For this, the medium was removed and the wells were washed 3 times with phosphate buffered saline solution (PBS), fixed with 4% glutaraldehyde and stained with 10% Giemsa. The cover slips were then mounted on a slide with Eukitt® embedding balm and observed under microscope. Images were acquired under Amscope T490B light microscope at X1,000 magnification.

### 2.9 Genes quantification and antimony-resistance markers expression rate

After sand flies infection, the parasites were isolated from the infected digestive tracts and maintained in culture on a free-drug medium to assess genes quantification and Sb(III)-resistance markers expression rate. Thus, 1 mL of each culture were centrifuged at 10,000 *g*, washed 3 times with 1X PBS and DNA was extracted from the pellet using Kit Isolate II genomic DNA®” (Bioline Meridian, USA) according to the supplier's recommendations then quantified with NanoDrop™ (ThermoFisher, France). In parallel, the same amount of parasite cultures was used for total RNA extraction. After centrifugation, the pellet was placed in TRIzol™ reagent (ThermoFisher, France) and stored at −80 °C. Total RNAs were extracted using RNA Purification Kit (ThermoFisher, France) according to the supplier's recommendations. Two real-time quantitative PCR (qPCR) experiments were performed, and the samples were analyzed in triplicates. To assess the copy number of specific resistance genes (Table 1), qPCR technique was applied on susceptible *L. major* (LIPA-WT) and its derivative resistant lines (SbIIIR-0.5 mM and SbIIIR-5.5 mM) before and after sand flies infection using the CFX OPUS 96® (BioRad, France). The experiment was carried out on a total volume of 20 μL. The final Mix included the SensiFAST® NO-ROX reagent (Bioline Meridian, France) at 1X, 400 nM of sense and antisense primers (Table 1) and 2 μL of the genomic DNA to be analyzed. The DNA of *Leishmania major* MHOM/PT/92/CRE26 was used as positive control. The glyceraldehyde-3-phosphate dehydrogenase (GAPDH) gene that codes for a glycolytic enzyme of *L. major*, was used as a reference gene to run the study (F): GAAGTACACGGTGGAGGCTG and (R): CGCTGATCACGACCTTCTTC).

**Table 1:** Sequence of qPCR primers used for amplification assay and expression genes involved in *Leishmania* antimony resistance.

Symbol	Genes	Reference sequence	sense	Sequence 5'→3′
*LmPRP1*	**pentamidine resistance protein 1 (PRP1)**	LMJFC_230007600	F	GAT-GAT-GCT-GTG-CTG-GAC-TG
			R	TCA-GCC-ACA-TCA-GCA-GGT-AA
*LmMRPA*	**ABC-thiol transporter**	XM_001683297.1	F	CAA-GAT-GCA-CCT-CGT-ACT-GC
			R	CAG-CTC-ACA-AAC-TCG-AGA-CG
*LmMDR1*	**p-glycoprotein (MDR1)**	XM_001686140.1	F	AAT-CCT-CTG-TTC-AGT-GCC-GA
			R	GAG-ATC-CAT-TGC-GAT-ACG-GC
*LmJBP1*	**DNA J-binding protein (JBP1)**	XM_001681269.1	F	CCG-CGC-GAT-GTG-ATG-ATA-TT
			R	GGT-AAG-TCG-CTT-CCA-GTC-CT
*LmAQP1*	**aquaglyceroporin (AQP1)**	XM_001684934.1	F	ACT-CAG-CTG-TAC-GTG-GAC-AA
			R	TCG-CTT-ACT-CTC-GTG-TTG-GT
*LmTDRX*	**thioredoxin - putative**	LMJFC_010007800	F	AAG-GAT-CTG-GAC-AGG-CTC-AC
			R	ATG-TCC-GTG-TTG-TTG-TCT-GC
*LmTXN1*	**tryparedoxin (TXN1)**	XM_003722147.1	F	TGA-GAA-GCA-CCA-CGA-TTC-GA
			R	TTA-GCG-CCT-CCA-CAA-TGT-TG
*LmTDR1*	**Thiol-dependent reductase 1**	LmjF.33.0240	F	GAG-TCG-CAG-CTG-ATT-GTT-CA
			R	CCA-CCT-CGT-ACT-CCT-TCT-CC
*LmPRX*	**Peroxiredoxin**	XM_001683274.1	F	ACT-ACG-GTG-TGC-TGA-TCG-AA
			R	GTC-ATT-GAT-CGT-CGA-GTG-GC

To assess the gene expression, real-time quantitative reverse transcription PCR (qRT-PCR) assay was performed in 20 μL reactions volume using the SensiFAST® NO-ROX reagent (Bioline Meridian, France) as previously described using CFX OPUS 96® (BioRad, France). To run the experiment, 1 μg of isolated total RNA was treated with DNase I, Amplification Grade® (Life Technologies®) then reverse transcribed with Superscript® VILO™ (Life Technologies®) following the manufacturer's recommendations. The qPCR cycling conditions were 95 °C for 2 min, followed by 30 cycles of 95 °C for 5 s, 60 °C for 10 s and 72 °C for 15 s. Primer amplification specificity was assessed by melting curve analysis. All samples were tested in triplicate, and the cycle threshold (Ct) was set at 0.1.

## 3 Results

### 3.1 Selection of antimony Sb(III)-resistant lines

After stepwise *in vitro* drug pressure, two antimony-resistant lines SbIIIR-0.5 mM and SbIIIR-5.5 mM were obtained from the *L. major* LIPA-WT strain, and were maintained *in vitro* under Sb(III)-drug pressure of 0.5 mM and 5.5 mM respectively to run the *in vivo* and *in vitro* assays with a maximum of two sub-passages to prevent parasites from losing their virulence as previously recommended (Bussotti et al., 2023).

### *3.2* L. major *Mon-*25 *in vivo fitness*

#### 3.2.1 Sand fly infection rates and parasite loads

**Fig. 1:**
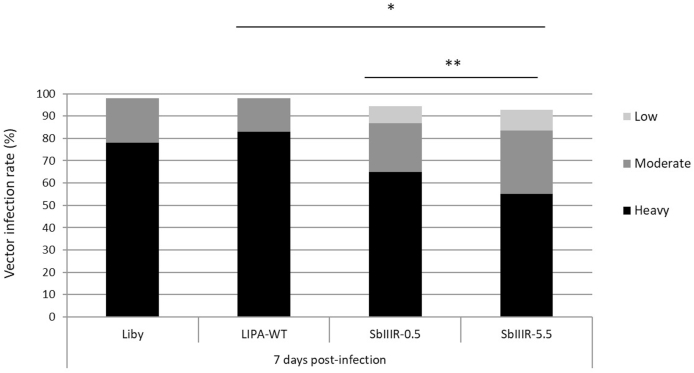
Infection rates of females *Ph. papatasi* infected with *L. major* wild-type strains (Liby and LIPA-WT) and Sb(III)-resistant lines (SbIIIR-0.5 mM and SbIIIR-5.5 mM) on day 7 p. i. Parasite loads were estimated under the light microscope: low infection <100 parasites per gut, moderate infections 100–1000 parasites per gut, heavy infection >1000 parasites per gut. Differences between the groups (not infected/light infection/moderate infection/heavy infection) were evaluated using the Chi-square test (χ2). The statistical differences are considered significant if *P_value_<0.05 and highly significant if **P_value_<0.01.

The development of the resistant and wild-type parasites in the infected sand flies was evaluated in a total of 90 dissected females per line to compare *i)* the infection rate and parasite loads, *ii)* the location of the parasites in the digestive tracts and *iii)* the morphological forms of the parasites. Two days post-infection (p.i.), the principal aim of the dissection was to assess the infection rate of blood-fed female sand flies and in this respect, there were no significant differences among the four strains and lines (*P*_*value*_ = 0.2, χ^2^ = 1.420, d. f. = 1); infection rates were above 95% in all groups (Fig. S1, Supplementary data). In late-stage infection on day 7 p. i (Fig. 1), infection rate of female sand flies remained high while the intensity of infection was different among the groups (*P*_*value*_ *=* 0.0422, χ^2^ = 22.42, d. f. = 3). In both wild-type strains (LIPA-WT and Liby), more than 70% of the infected females presented heavy parasite loads which were less represented in both resistant lines. Besides, the parasite load in females infected with SbIIIR-0.5 mM parasites was significantly higher than the females infected with SbIIIR-5.5 mM line (*P*_*value*_ *=* 0.0098, χ^2^ = 34.62, d. f. = 3), (Fig. 1).

#### *3.2.2* *Location of parasites in* Ph. papatasi *digestive tracts*

**Fig. 2:**
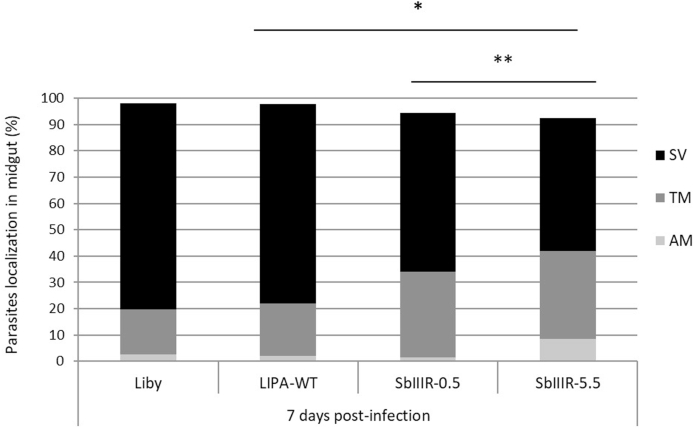
Localization of L. *major* wild-type (Liby and LIPA-WT) and Sb(III)-resistant parasites (SbIIIR-0.5 mM and SbIIIR-5.5 mM) in guts of *Ph. papatasi* on day 7 p. i., **SV:** stomodeal valve, **TM:** thoracic midgut, **AM:** abdominal midgut. Differences between the groups (AM, TM, SV) were evaluated using the Chi-square (χ2) test. The statistical differences are considered significant if *P_*value*_<0.05 and highly significant if **P_*value*_*<*0.01.

Two days p. i., for each batch of infected sand flies (LIPA-WT, Liby, SbIIIR-0.5 mM and SbIIIR-5.5. mM), 100% of the parasites were localized in the abdominal midgut of sand flies vector, inside the peritrophic matrix that surrounds the blood meal. On day 7 p. i., the blood meal was completely digested, and the parasites moved from the abdominal midgut to the thoracic midgut (Fig. S2, Supplementary data). The stomodeal valve (SV) of infected female sand flies was heavily colonized with parasites, a sign of mature infections and a prerequisite for parasites transmission. The rate of SV colonization in infected females was significantly higher in the wild-type strains (LIPA-WT and Liby) compared to both resistant-lines (*P*_*value*_ = 0.00325, χ^2^ = 28.65, d. f. = 3), there was also a significant difference between the two resistant lines SbIIIR-0.5 mM and the SbIIIR-5.5 mM lines (*P*_*value*_ = 0.0001, χ^2^ = 45.12, d. f. = 2) (Fig. 2).

#### 3.2.3 Morphology of parasites inside the sand fly digestive tracts

**Fig. 3:**
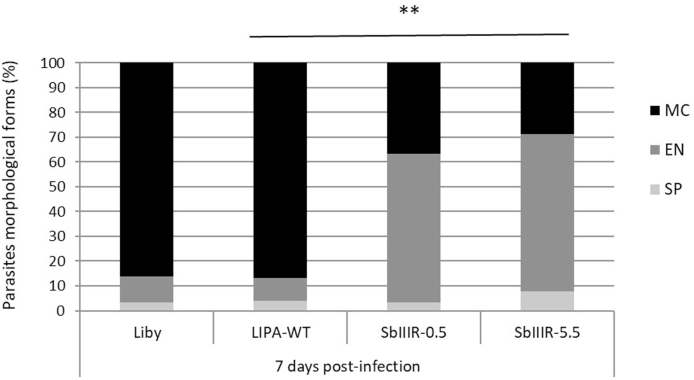
Morphological forms of *L. major* LIPA-WT observed in infected *Ph. papatasi* at day 7 p. i. Parasites on gut smears of infected sand flies were fixed with methanol and stained with Giemsa. Scale bar = 10 μm. **SP:** short promastigote, **EN:** elongated nectomonad, **MC:** metacyclic promastigote.

On day 7 p. i., four different morphological forms of parasites were observed: short promastigotes (SP), elongated nectomonads (EN), metacyclic promastigotes (MC) (Figure S3, Supplementary data) and rounded haptomonads. The relative representation of these morphological forms significantly differed between susceptible and resistant parasites (*P*_*value*_ *=* 0.0008, χ^2^ = 38.56, d. f. = 3). While the elongated nectomonads prevailed in both resistant lines, metacyclic forms were dominant in both-wild type strains (Fig. 3). The relative representation of haptomonads was not evaluated because they remain attached to the stomodeal valve and are therefore underrepresented on the gut smears.

The results presented in this study showed clearly that the resistance to Sb(III) does not influence the early-stage development of *L. major* in sand flies as the infection rate was almost 100% in females infected with both susceptible or Sb(III)-resistant lines on day 2 p. i. In mature infections, heavy parasite loads were significantly more represented in female sand flies infected with both WT strains (LIPA-WT and Liby). In these groups, also the colonization of the SV was more often developed and metacyclic forms were more represented compared to females infected with Sb(III)-resistant lines (SbIIIR-0.5 mM and SbIIIR-5.5 mM). Therefore, a negative correlation was observed between the resistance level and the quality of parasite development in the vector's gut. The more *Leishmania* parasites were resistant, the less they colonized the SV and less heavy infections and metacyclic forms were observed.

### *3.3* In vitro *fitness of parasites isolated from infected sand flies*

The antimony-susceptibility, promastigote growth curves, infection rates and parasite loads in macrophages were studied in LIPA-WT, SbIIIR-0.5 and SbIIIR-5.5 mM parasites recovered from infected guts of *Ph. papatasi*. From 10 guts dissected per each *L. major* line, 2 guts free from any fungal contaminants were studied.

#### 3.3.1 Parasites Sb(III)-susceptibility

Standard susceptibility assays were performed on promastigote forms to assess the Sb(III)-susceptibility of each parasite lines: LIPA- WT, SbIIIR-0.5 mM and SbIIIR-5.5 mM, before and after their passage through *Ph. papatasi* digestive tract (Table 2).

**Table 2:** Susceptibility of L. *major* lines to Sb(III) drug before and after passage in females *Ph. papatasi.* Tests were running in duplicate and results were expressed as IC_50_ values that were determined by a nonlinear regression using ICEstimator website 1.2 version, and expressed as mean IC_50_ value (μM) ± standard error of the mean (SEM).

*Leishmania* promastigotes	IC_50_ ± SEM (μM)	
	Before infection	After infection
LIPA-WT	10.76 ± 2.38	2.55 ± 0.60
SbIIIR-0.5 mM	50.85 ± 4.65	1593 ± 100.08
SbIIIR-5.5 mM	250.8 ± 100.8	3342.31 ± 250.64

It should be noted that after passage of the parasites in sand fly guts, all three *Leishmania* lines were maintained in Sb(III)-free culture medium before calculation of the IC_50_ values. Results showed that the IC_50_ of the LIPA-WT strain decreased approximately 4-folds while the IC_50_ of both resistant lines increased 30-fold and 14-fold (SbIIIR-0.5 mM and SbIIIR-5.5 mM respectively) (Table 2). This would suggest that the environment inside the sand fly guts selected the highly resistant parasites under the vector bottle neck effect.

#### 3.3.2 Promastigote growth curves

**Fig. 4:**
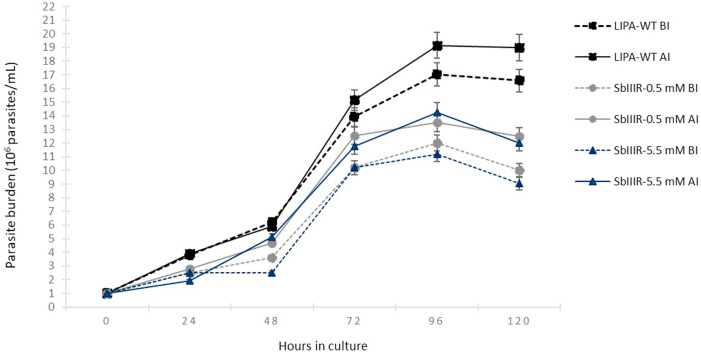
Promastigote growth curves of *L. major* (LIPA-WT) and of its Sb(III)-resistant lines (SbIIIR-0.5 mM and SbIIIR-5.5 mM). **BI:** before infection, **AI:** after infection. Parasite density was determined in duplicate every 24 h (24 h–120 h). Data were analyzed with GraphPad Prism software version 7.05 (GraphPad Software Inc.). Two-way ANOVA followed by *Dunnett's* multiple comparisons test was used. Results were expressed as mean ± SD and considered significant if *P*_*value*_ < 0.05.

Growth curves of the LIPA-WT and the two selected resistant lines were compared before and after passage through sand fly guts (Fig. 4).

LIPA-WT line as well as both Sb(III)-resistant lines reached their peak of growth after 96 h in culture. Direct microscopic observation of the parasites in culture between 96 h and 120 h showed that the parasites reached their maximum capacity of multiplication and started to slowly lose their mobility which corresponds to the growth stationary phase. After 120 h in culture, the parasites started to transform into rounded forms corresponding to parasite apoptosis point. Although the multiplication *in vitro* was slightly enhanced post-passage through sand flies (LIPA-WT = from 17.01 × 10^6^ to 19.15 × 10^6^ parasites/mL, SbIIIR-0.5 mM = from 12.01 × 10^6^ to 13.50 × 10^6^ parasites/mL and SbIIIR-5.5 mM = from 11.02 × 10^6^ to 14.25 × 10^6^ parasites/mL), the differences were not significant (*P*_*value*_ = 0.088). Thus, these data suggest that the passage in the sand fly digestive tracts did not significantly change the *in vitro* multiplication and growth capacity of the parasites.

#### 3.3.3 Development of L. *major* WT and Sb(III)-resistant derivative lines in macrophages RAW 264.7

**Fig. 5:**
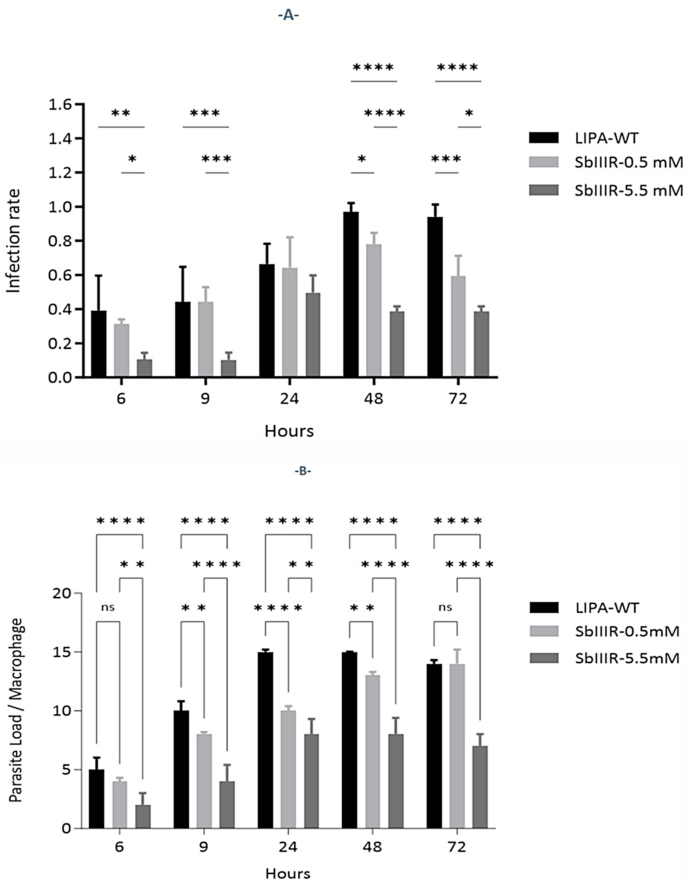
Infection rate and parasite load of *L. major* LIPA-WT, SbIIIR-0.5 mM and SbIIIR-5.5 mM in murine macrophages RAW 264.7. (A) Infection rate = number of infected macrophages/number of total macrophages, (B) Parasite load/Macrophage = number of amastigotes/macrophages. The assay was assessed for 72 h. Parasites were fixed with paraformaldehyde (PFA), stained with 10% Giemsa then observed under light microscopy X1,000. Three experiments were carried out independently at 37 °C under 5% CO2 in a dark environment. Kinetics was carried at 6 h, 9 h, 24 h, 48 h and 72 h then (A) the number of infected macrophages and (B) the number of parasites that infected the macrophages were counted for each *Leishmania* lines. Two-way ANOVA followed by *Tukey's* test was used for multigroup comparisons. Results were expressed as mean ± SD and were based on three independent experiments (*P_*value*_< 0.05, **P_*value*_< 0.01, ***P_*value*_< 0.001 and ****P_*value*_< 0.0001).

The Sb(III)-resistant parasites (SbIIIR-0.5 mM and 5.5 mM) were compared to the LIPA-WT strain in i) the speed of entry into macrophages and morphology of intra-macrophage amastigote forms (Figure S4, Supplementary data), ii) the infection rate (the representation of infected macrophages) (Fig. 5. A), and iii) parasite loads in macrophages (Fig. 5. B).

In all three *L. major* lines, the promastigote parasites were able to differentiate into amastigote forms in RAW 264.7 macrophages. Under light microscopy, the amastigote forms showed an average diameter of 1.45 μm (LIPA-WT), 2.88 μm (SbIIR-0.5 mM) and 2.5 μm for SbIIIR-5.5 mM (data not shown). The LIPA-WT parasites and the resistant derivative SbIIIR-0.5 mM parasites were able to penetrate the cells after 6 h in contact while the SbIIIR-5.5 mM-resistant line spent an average of 10 h to infect the macrophages (LIPA-WT/SbIIIR-5.5 mM: *P*_*value*_< 0.01; SbIIIR-0.5mM/SbIIIR-5.5 mM: *P*_*value*_< 0.05).

The maximum infection rate of RAW 264.7 cells was reached after 48 h for LIPA-WT and SbIIIR-0.5 mM parasites whereas it required 24 h for SbIIIR-5.5 mM line to infect the macrophages (Fig. 5. B). However, the infection rate of SbIIIR-5.5 mM parasites remained significantly lower than in LIPA-WT and SbIIIR-0.5 mM lines.

In addition, SbIIIR-5.5 mM showed also the lowest number of amastigotes per macrophage: between 10 and 15 amastigotes were counted on RAW 264.7 macrophages for LIPA-WT and SbIIIR-0.5 mM lines and between 5 and 10 for SbIIIR-5.5 mM resistant parasites.

### 3.4 Study of antimony-resistance biomarkers

The targeted resistance genes (Table 1) were analyzed using the quantitative real-time PCR (qPCR) to compare (***i***) the amplification rate (gene copy number) before and after the passage of the parasites in sand flies, (***ii***) the amplification rate within and between the three L. major lines LIPA-WT, SbIIIR-0.5 mM and SbIIIR-5.5 mM and *(****iii****)* the expression rate of each targeted resistance marker.

**Fig. 6:**
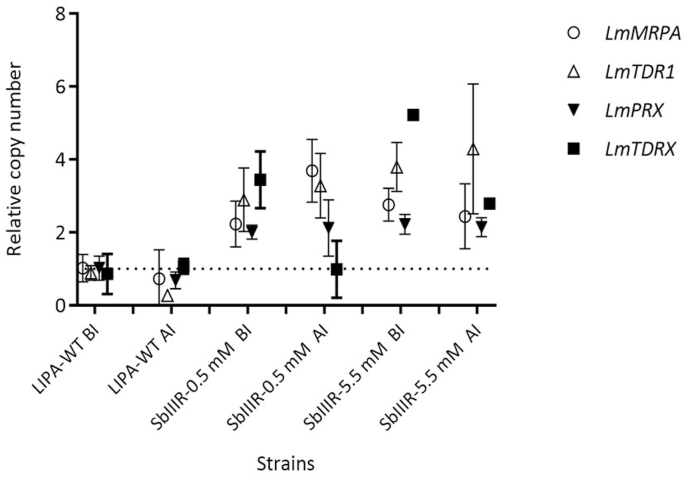
Targeted gene copy number of *L. major* LIPA-WT, SbIIIR-0.5 mM and SbIIIR-5.5 mM lines after *in vitro* drug pressure and after passage in *Ph. papatasi* digestive tracts. The copy number was expressed as a ratio of *LmTDR1*, *LmTDRX*, *LmPRX* or *LmMRPA* gene to *GAPDH* gene and the data of reference strain *Leishmania major* to obtain the ΔΔCt. Data were expressed as the mean ± SD of three independent experiments. **BI:** before infection, **AI:** after infection. Two-way ANOVA followed by *Bonferonni's* test was used for multigroup comparisons. Results were expressed as mean ± SD and were based on three independent experiments.

For this, we presented only the markers that showed noticeable changes in their copy numbers (Table S1, Supplementary data). For the susceptible LIPA-WT strain, either maintained in free-drug medium or after its passage through sand fly guts, we noticed that the copy number of three genes *LmMRPA, LmPRX* and *LmTDRX* did not change and have the same amplification level except for *LmTDR1* gene which presented a slight decrease of its copy number (Fig. 6; Table S1, Supplementary data). Regarding both Sb(III)-resistant lines, the copy number of *LmTDRX* gene decreased two fold after the passage of the resistant parasites in the sand fly guts. The copy number of *LmTDR1* slightly increased after infection of sand flies, *LmMRPA* was amplified only for the SbIIIR-0.5 mM line. The *LmPRX* gene copy number remained unchanged in the three parasite lines (Fig. 6; Table S1, Supplementary data).

When we compared the amplification rate (differences in copy numbers before and after the passage through sand fly guts) of four markers: *LmTDR1, LmTDRX, LmMRPA*, and *LmPRX* genes between LIPA-WT and Sb(III)-resistant lines, we noticed a significant difference in the relative copy number except for the couple LIPA-WT *vs* SbIIIR-0.5 mM for which *LmPRX* and *LmTDRX* genes respectively: before and after passage of the parasites in sand fly gut; did not show significant differences in their relative gene copy number (Fig. 6; Table S2, Supplementary data).

After the passage of the three *Leishmania* lines (WT and Sb(III)-resistant) in the sand fly digestive tract, the copy number of *LmTDRX* gene decreased significantly in both resistant lines compared to LIPA-WT line while *LmMRPA* gene increased significantly its gene copy numbers in SbIIIR-0.5 mM resistant line. No differences in genes copy number were detected for the two other genes: *LmPRX* and *LmTDR1*. This suggests that *LmTDRX* gene would not be involved during the vector phase of *Leishmania* life-cycle. It is important to notice that *LmTDR1* and *LmTDRX* represent genes involved in the detoxification pathway in *Leishmania* parasites metabolism while *LmMRPA* gene code for ABC-thiol transporter involved in the sequestration of metal as thiol conjugate into a vacuole.

**Fig. 7:**
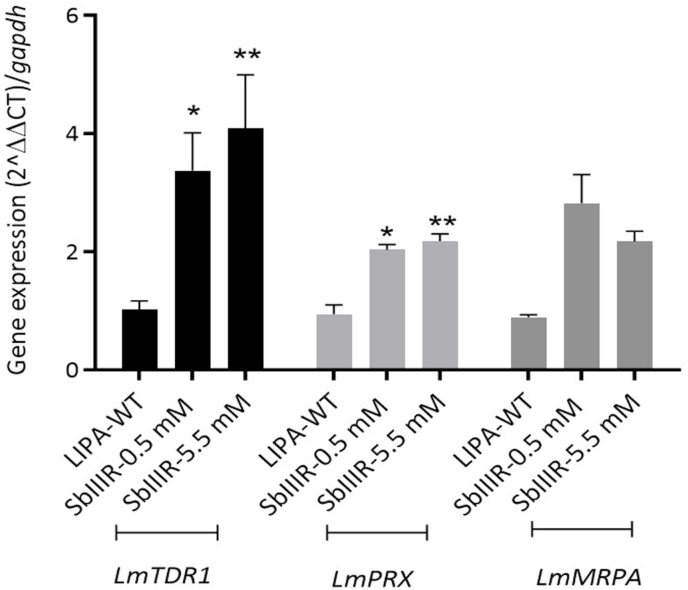
Graphical representation of *LmTDR1*, *LmPRX* and *LmMRPA* gene expression normalized on *GAPDH* gene. The assay was ran on LIPA-WT, SbIIIR-0.5 mM and SbIIIR-5.5 mM lines after passage on infected *Ph. papatasi* digestive tracts. The gene expression level was expressed as a ratio of *LmTDR1*, *LmPRX* or *LmMRPA* gene, and mRNA level to *GAPDH* mRNA level. Data were expressed as the mean ± SD of three independent experiments. Two-way ANOVA followed by *Tukey's* test was used for multigroup comparisons. Results were expressed as mean ± SD and were based on three independent experiments (*P_*value*_ < 0.05, **P_*value*_ < 0.01).

On the other hand, the evaluation of genes expression rate showed that three genes *LmMRPA, LmTDR1* and *LmPRX* were significantly expressed *in vivo* in both Sb(III)-resistant lines compared to the LIPA-WT strain despite the absence of drug. *LmPRX* gene is expressed 2 folds in both resistant lines while *LmMRPA* and *LmTDR1* genes were expressed 3 and 4 folds respectively in SbIIIR-0.5 and SbIIIR-5.5 mM lines compared to LIPA-WT (Fig. 7).

It appears that these genes retained the increased copy number during drug-pressure and after the passage in the sand fly digestive tracts, which seems normal given that they code for detoxifying proteins necessary to counter the action of drugs but also the hostile environment of the sand fly gut such as digestive enzymes and immune system.

## 4 Discussion

This study aimed to assess the impact of Sb(III)-resistance on the development of *L. major in vivo* in the sand fly and *in vitro* by comparing different biological parameters such as *i)* parasite load, location and metacyclogenesis in sand flies, *ii) in vitro* growth capacity, iii*)* infectiousness to macrophages, i*v)* drug susceptibility and *v)* the expression rate of the resistant markers.

During the vector phase of the life-cycle, *Leishmania* parasites must *i)* resist to the action of sand fly proteolytic digestive enzymes*, ii)* escape the peritrophic matrix and *iii)* attach to the midgut epithelium to prevent excretion via defecation by the sand fly, migrate anteriorly and colonize the stomodeal valve (Kamhawi, 2006; Dostálová and Volf, 2012). Here in our study, whatever *L. major* line, the parasites were able to survive, colonize the stomodeal valve and differentiate into infective forms (metacyclic promastigotes) inside the sand fly guts despite the detected differences in representation of observed parasite forms. In addition, the parasites did not lose their fitness and the resistance was stable after the passage of the two resistant-lines through sand flies. The same results were reported for the couples Sb(III)-resistant *L. infantum/Lutzomya (Lu.) longipalpis* and Sb(III)-resistant *L. infantum*/*Ph. perniciosus* (Seblova et al., 2014) as well as paramomycin-resistant *L. infantum*/*Lu. longipalpis* and *Ph. perniciosus* and paramomycin-resistant *L. donovani*/*Ph. argentipes* (Hendrickx et al., 2020a, Hendrickx et al., 2020b). These results suggest that there is no general link between the resistance and the loss of fitness in *Leishmania* parasites in sand flies so that many resistant lines are able to spread and circulate in nature and thus threatening human and animal health. Nevertheless, the development of resistant *Leishmania* lines in sand fly depends on the type of drug and parasite species, since the development of miltefosine-resistant *L. infantum* was significantly impaired in *Ph. perniciosus* and *Lu. longipalpis* (Van Bockstal et al., 2019).

Another important and amazing point was the inverse effect observed in *L. major* lines after their passage through the vector: the susceptible phenotype of WT line became more susceptible while both resistant lines became more resistant. This could be explained by the hostile environment existing in the vector gut which exerts pressure on the different *Leishmania* clones or sub-populations composing each of the three *L. major* lines, thus by eliminating some sub-populations under the phenomena of bottle neck, recently reported in *L. donovani* strain after its transition in *Ph. orientalis* (Bussotti et al., 2023). This phenomenon could be caused by different parameters *i)* the small number of parasites ingested during a sand fly blood meal, *ii)* the potential elimination of parasites by toxic products during blood meal digestion, or *iii)* their failure to attach to the sand fly midgut that leads to parasites excretion via defecation of the digested blood meal (Bussotti et al., 2023; Pruzinova et al., 2018).

Moreover, the ability of *Leishmania* parasites to amplify the copy number of specific genes and to use the advantage of these extra copies may increase the levels of gene products and their drug-resistance phenotype (Ponte-Sucre et al., 2013). Moreover, the amplification of the *LmMRPA* gene obtained during this study is in line with previous findings. Leprohon et al. (2009) demonstrated that *L. infantum* KOs lines for *MRPA* gene restored the susceptibility to antimonials, while Douanne et al. (2020) observed that in case of complete deletion of the gene, other transporters can be involved in drug resistance mechanism.

In addition, it was not surprising to see an increase in the copy number of some genes related to the detoxification pathway in the resistant lines since it is well known that one of the unique features of *Leishmania* parasites is “Gene plasticity” and their ability to alter the copy number of individual genes or in some cases; to alter a group of genes, chromosomes and even the entire genome (Ponte-Sucre et al., 2013). Indeed, the detoxification pathway is essential for *Leishmania* parasite to escape the host defense mechanism and to survive the drug treatment. Thus, many proteins are expressed to activate this pathway for the survival of the parasite within the host and already reported in other parasites and yeast species such as *Trypanosoma brucei brucei and Saccharomyces cerevisiae* in response to oxidative stress (Pedrajas et al., 1999; Reckenfelderbäumer et al., 2000). The thiol-dependent reductase 1 (TDR1) of *Leishmania* sp. has been implicated in parasite-host interaction. Like other proteins in the glutathione/trypanothione-based redox system, this gene allows the reduction of Sb(V) to Sb(III) and is over-expressed in antimony-resistant parasites (Denton et al., 2004; Nateghi-Rostami, Tasbihi & Darzi, 2022). However, these proteins have not yet been studied to understand their implication and specific role in the survival of *Leishmania* parasites during the vector phase of their life-cycle. For this, a complete genome sequencing of various *Leishmania* species could help to identify and validate new targets as resistance biomarkers to better understand the apparition of resistance mechanisms as well as to identify new therapeutic targets against *Leishmania* parasite (Ponte-Sucre et al., 2013).

## 5 Conclusion & perspectives

Drug resistance becomes the major obstacle to the treatment, prevention and eradication of parasitic or bacterial diseases for humans in the absence of efficient vaccine (Sereno et al., 2012; Dujardin et Decuypere, 2013). Here, we established a laboratory experimental model *Ph. papatasi/L. major*, trying to be mimetic of parasite life cycle in the natural conditions which provided suitable indications about the potential transmission of parasite populations resistant to trivalent antimony by the sand fly in controlled conditions. The results presented in this study showed that *Ph. papatasi* could promote circulation of *L. major* Sb(III)- resistant parasites in the field. More disturbingly, our data suggest that the selective pressures from the vector may even enhance antimony resistance. Further experiments are needed to confirm this surprising finding. Moreover, studies using new molecular tools as WGS, NGS and CRISPR-cas9 will allow to better understand *i)* the role of *LmMRPA* and *LmTDR1* during the sand fly stage of parasites life-cycle *ii)* the risks of transmission of these drug-resistant parasites in the field to anticipate the evolution of the epidemiological situation in areas being endemic with zoonotic cutaneous leishmaniasis caused by *L. major* MON-25.

## Authors contribution

Conceptualization: N.M, Z.H., S.C., P.M.L, K.E.B., J.S., B.V.; Parasites analyses: N.M., S.C., N.I., M.L., A.M.; Sand fly experiments: J.S., B.V., N.M., K.E.B.; Writing original draft: N.M., K.E.B., S.C., PM.L.; Editing: J.S., P.V., P.M.L., Z.H. All authors have read and agreed to the published version of the manuscript.

## Funding

This study was partially sustained by the collaboration between Institut Pasteur d’Algérie and Charles University; funded by the European Union's Horizon 2020 RIIP-LeiSHield MATI-RISE research and innovation program under the Marie Skłodowska Curie grant agreement No. 778298 supporting the missions of N.M.; J.S. and P.V. were partially funded by the Czech 10.13039/100009950Ministry of Education, ERD funds, project CePaViP (CZ.02.1.01/0.0/0.0/16_019/0000759).

## CRediT authorship contribution statement

**Nalia Mekarnia:** Writing – original draft, Visualization, Validation, Methodology, Investigation, Formal analysis, Data curation, Conceptualization. **Benallal Kamal-Eddine:** Writing – original draft, Methodology, Investigation, Formal analysis, Data curation, Conceptualization. **Jovana Sádlová:** Writing – original draft, Validation, Supervision, Resources, Methodology, Investigation, Formal analysis, Data curation, Conceptualization. **Barbora Vojtková:** Writing – original draft, Visualization, Validation, Supervision, Resources, Methodology, Investigation, Formal analysis, Data curation. **Aurélie Mauras:** Methodology, Data curation. **Nicolas Imbert:** Methodology. **Maryline Longhitano:** Methodology. **Zoubir Harrat:** Writing – original draft, Resources, Project administration, Investigation, Funding acquisition, Conceptualization. **Petr Volf:** Writing – original draft, Validation, Supervision, Resources, Investigation, Funding acquisition, Formal analysis, Data curation, Conceptualization. **Philippe M. Loiseau:** Writing – original draft, Validation, Supervision, Funding acquisition, Formal analysis, Conceptualization. **Sandrine Cojean:** Writing – original draft, Validation, Supervision, Resources, Methodology, Formal analysis, Data curation, Conceptualization.

## Declaration of competing interest

The authors declare that there is no conflict of interest regarding the publication of this article.
