# Supplementary material for: Effect of Phlebotomus papatasi on the fitness,infectivity and antimony-resistance phenotype of antimony-resistant Leishmania major Mon-25
Source: Int J Parasitol Drugs Drug Resist. 2024 Jun 24;25:100554. doi: 10.1016/j.ijpddr.2024.100554 (PMC11261056; doi:10.1016/j.ijpddr.2024.100554)
Supplement: Multimedia component 1 [file mmc1.docx]

*Supplementary data*

**Effect of *Phlebotomus papatasi* on the fitness, infectivity, and antimony-resistance phenotype of antimony-resistant *Leishmania major* Mon-25**

Nalia MEKARNIA^1,2^, Kamal-Eddine BENALLAL^3,4^, Jovana SÁDLOVÁ^3^, Barbora VOJTKOVÁ^3^, Aurélie MAURAS^2^, Nicolas IMBERT^2,5^, Maryline LONGHITANO^2,6^, Zoubir HARRAT^7^, Petr VOLF ^3^, Philippe M. LOISEAU^2*^, Sandrine COJEAN^2,5,6^

^1^ UR 7510 ESCAPE - USC Anses, School of Pharmacy, Université de Reims Champagne-Ardenne, Pôle Santé, 51100 Reims, France.

^2^ UMR 8076 BioCIS, CNRS, Université Paris-Saclay, 91400 Orsay, France.

^3^ Department of Parasitology, Faculty of Sciences, Charles University, Vinicna 7, Prague, Czech Republic.

^4^ Laboratory of Arboviruses and Emergent Viruses, Institut Pasteur d’Algérie, 16047 Algiers, Algeria.

^5^ UMR BIPAR, Laboratory of Animal Health, Anses, INRAe, EnvA, 94700 Maisons-Alfort, France

^6^ National Malaria Reference Center, AP-HP, Hôpital Bichat Claude Bernard, 75018 Paris, France

^7^ Algerian Academy for Science and Technology, Villa Rais Hamidou, Chemin Omar Kachkar, El Madania, Algiers, Algeria.

* Corresponding author : Prof. Philippe LOISEAU. Chimiothérapie Antiparasitaire, UMR 8076 CNRS BioCIS. Bâtiment Henri Moissan (BPC), Bureau 4621. At : Université Paris-Saclay, Faculté de Pharmacie, 17 Avenue des Sciences - 91400 Orsay, France. Email : [philippe.loiseau@universite-paris-saclay.fr](mailto:philippe.loiseau@universite-paris-saclay.fr)

**Supplementary data to Results**


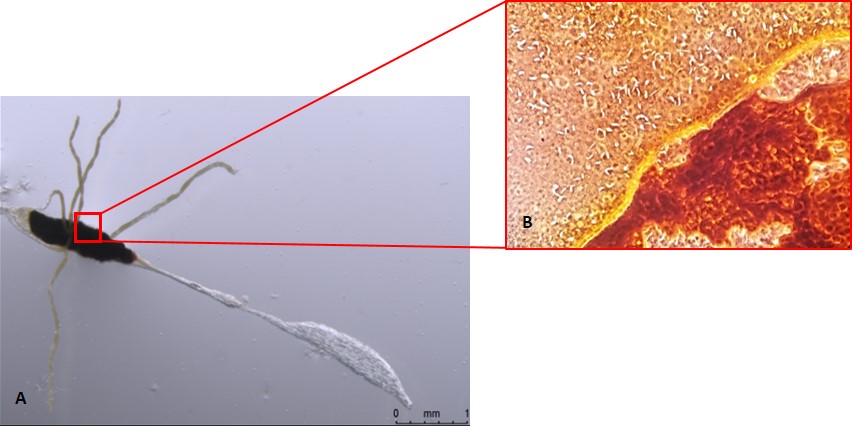


**Figure S1. Midgut of *Ph. papatasi* engorged female 2 days p.i. with detail of the dissected gut, observed in light microscopy.** Scale bar = 1 mm. **A:** complete digestive tract with blood meal**, B:** detailed section of the dissected gut. Parasites evaluation is difficult due to the presence of a large amount of red blood cells.

**
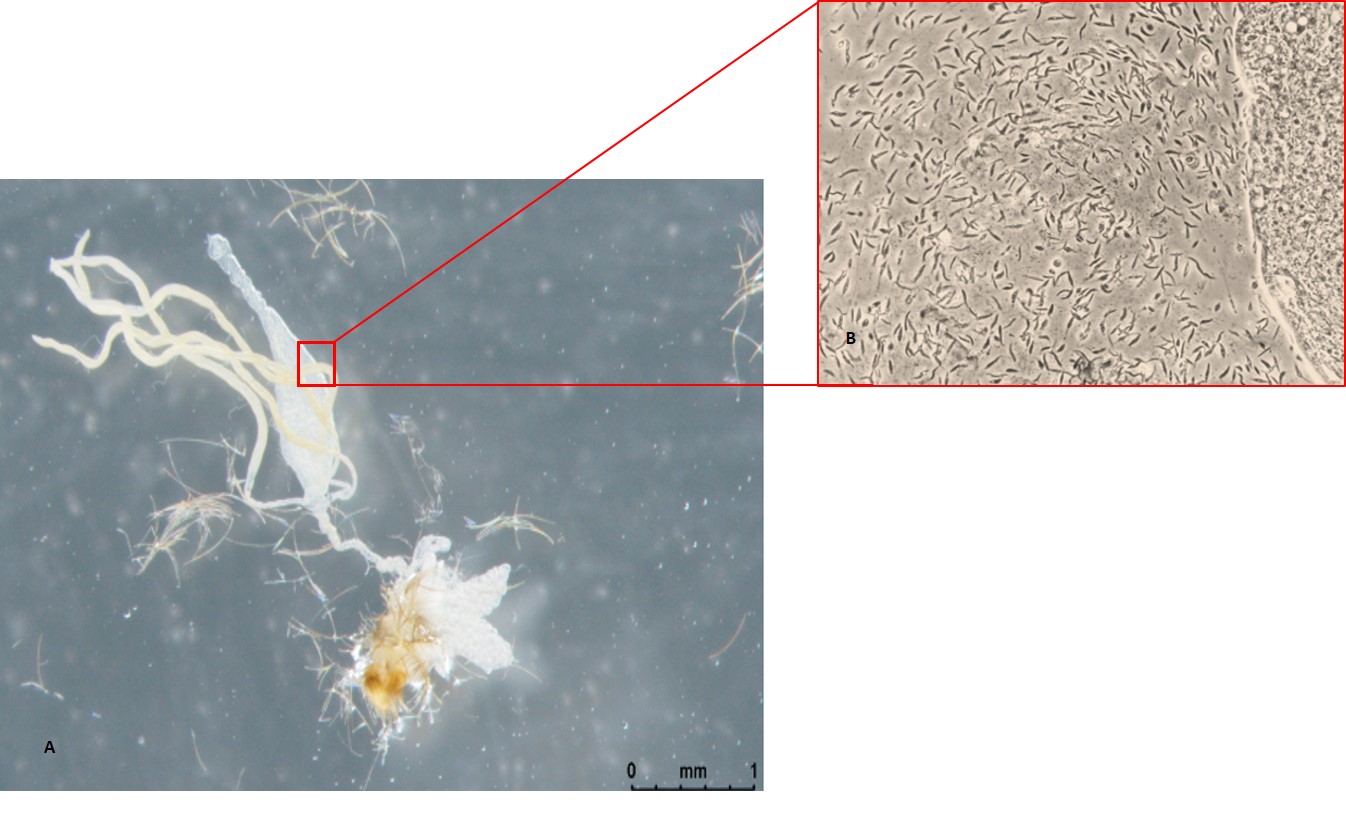
**

**Figure S2. Midgut of *Ph. papatasi* infected female 7 days p.i. with detail of the dissected gut, after total digestion of the blood meal, observed in light microscopy.** Scale bar = 1 mm. **A:** complete digestive tract with no blood meal**, B:** detailed section of the dissected gut. Parasites evaluation is easy due to the absence of red blood cells.


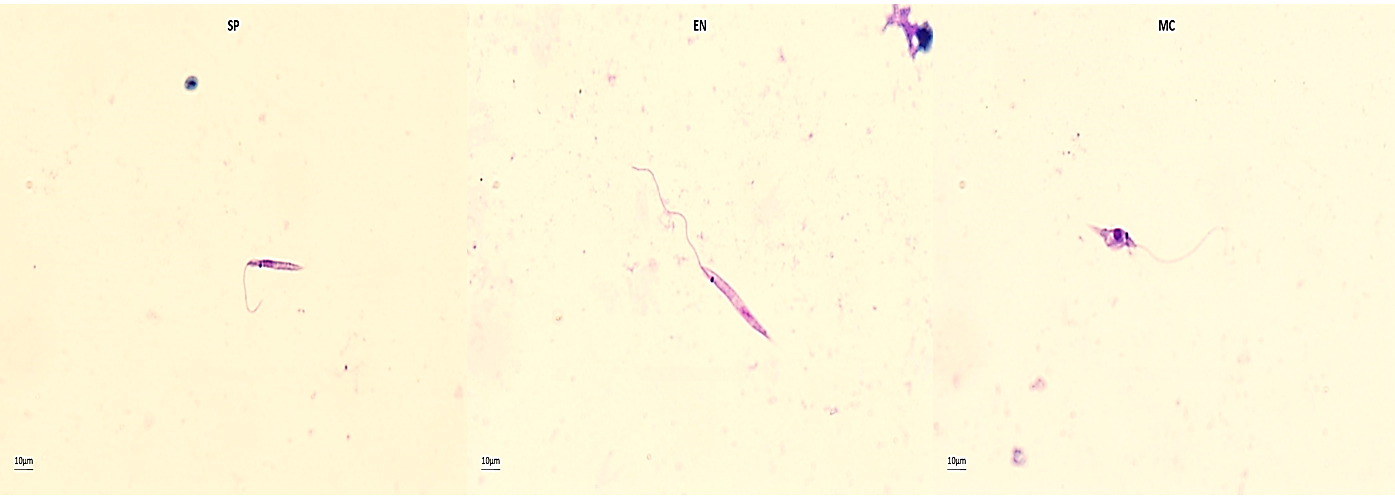


**Figure S3. Morphological forms of *L. major* LIPA-WT observed in infected *Ph. papatasi* at day 7 p.i**. Parasites on gut smears of infected sand flies were fixed with methanol and stained with Giemsa. Scale bar = 10 μm. **SP:** short promastigote, **EN:** elongated nectomonad, **MC:** metacyclic promastigote.


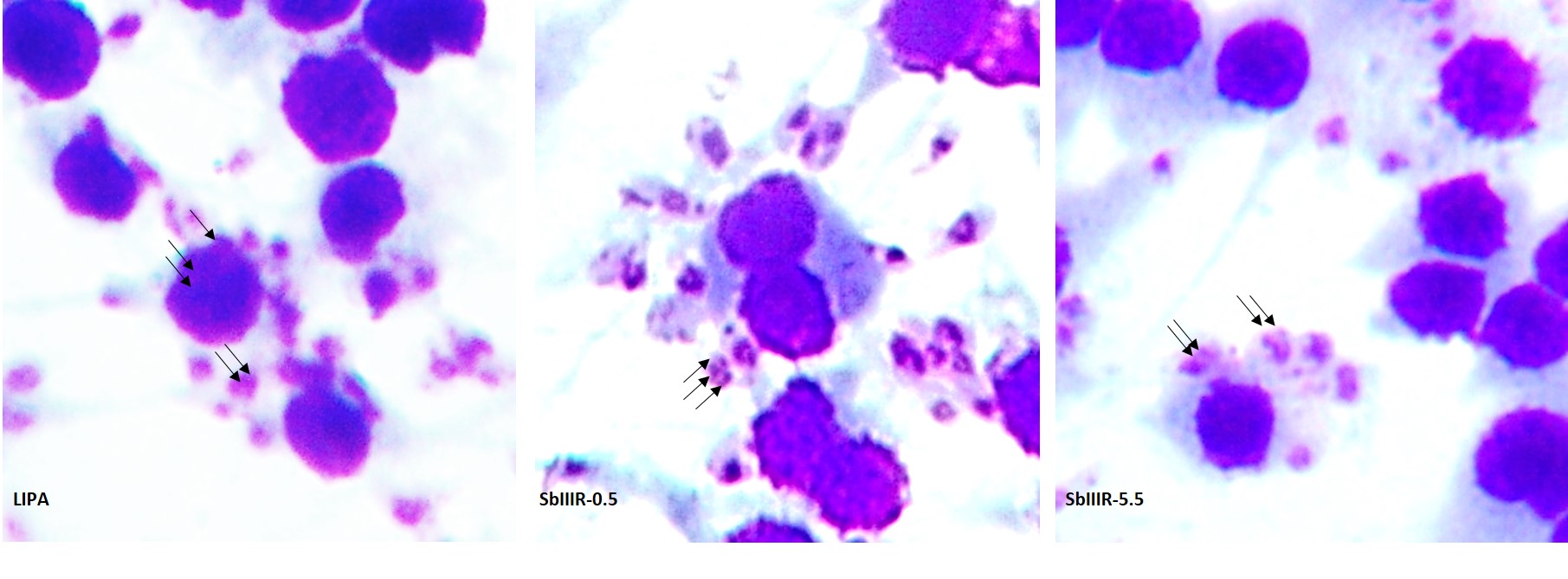


**Figure S4.** **Capacity of differentiation and morphological aspect of intramacrophage amastigote forms of *L. major* LIPA-WT, SbIIIR-0.5 mM and SbIIIR-5.5 mM lines on *in vitro* culture.** The assay was assessed for 72 hours. Parasites were fixed with paraformaldehyde (PFA), stained with 10% Giemsa then observed under an optical microscope x1,000. Three experiments were carried out independently at 37°C under 5% CO_2_ in a dark environment.

**Table S1.** **Gene copy numbers of *L. major* LIPA-WT, SbIIIR-0.5 mM and SbIIIR-5.5 mM lines before and after passage in *Ph. papatasi* digestive tracts.** The experiments were performed in duplicate and the results are expressed as mean target genes copy number ± standard error of the mean (SEM).

|  |  |  | | *Lm* genes copy number | | | | | |  |
| --- | --- | --- | --- | --- | --- | --- | --- | --- | --- | --- |
| Target genes | LIPA-WT | | |  | SbIIIR-0.5 mM | |  | SbIIIR-5.5 mM | |  |
|  | *In vitro* drug pressure endpoint | | Post  infection | *P_value_* | *In vitro* drug pressure endpoint | Post  infection | *P_value_* | *In vitro* drug pressure endpoint | Post  infection | *P_value_* |
| *LmMRPA* | 1.02 ± 0.38 | | 0.73 ± 0.79 | 0.9742 | 2.23 ± 0.63 | 3.69 ± 0.86 | **0.0030** | 2.76 ± 0.45 | 2.44 ± 0.89 | 0.9605 |
| *LmTDRX* | 0.86 ± 0.55 | | 1.07 ± 0.22 | 0.9940 | 3.44 ± 0.78 | 0.99 ± 0.78 | **0.0001** | 5.22 ± 0.10 | 2.79 ± 0.03 | **0.0001** |
| *LmTDR1* | 0.89 ± 0.20 | | 0.27 ± 0.03 | 0.5893 | 2.89 ± 0.87 | 3.28 ± 0.88 | 0.9115 | 3.79 ± 0.67 | 4.29 ± 1.78 | 0.7825 |
| *LmPRX* | 1.02 ± 0.33 | | 0.69 ± 0.23 | 0.9550 | 2.01 ± 0.19 | 2.12 ± 0.77 | 0.9997 | 2.22 ± 0.27 | 2.14 ± 0.26 | 0.9999 |

**Table S2.** **Comparison of the gene amplification rates of *LmMRPA*, *LmTDRX, LmTDR1*, and *LmPRX* resistance genes between *L. major* LIPA-WT *vs* SbIIIR-0.5 mM couple and LIPA-WT *vs* SbIIIR-5.5 mM couple, before and after passage in *Ph. papatasi* digestive tracts**. The experiments were performed in duplicate and the results are expressed as *P_values_*.

| Gene amplification rates (*P_values_*) | | | | | |  | |  |
| --- | --- | --- | --- | --- | --- | --- | --- | --- |
| Target genes | LIPA-WT *vs* SbIIIR- 0.5mM | | LIPA-WT *vs* SbIIIR-5.5mM | | | |  |  |
|  | *In vitro* drug pressure endpoint | Post  infection | *In vitro* drug pressure endpoint | Post  infection | |  | |  |
| *LmMRPA* | 0.0243 | 0.0001 | 0.0002 | | 0.0001 | |  | |
| *LmTDRX* | 0.0001 | **0.9999** | 0.0001 | 0.0002 | |  | |  |
| *mTDR1* | 0.0001 | 0.0001 | 0.0001 | 0.0001 | | |  |  |
| *LmPRX* | **0.1099** | 0.0039 | 0.0262 | 0.0033 | |  | |  |
